# Association between domains of quality of life and patients with Klinefelter syndrome: a systematic review

**DOI:** 10.1530/EJE-21-1239

**Published:** 2022-05-31

**Authors:** Brien Mehmet, Steve Gillard, Channa N Jayasena, Sofia Llahana

**Affiliations:** 1 School of Health Sciences, City, University of London, London, UK; 2 Section of Investigative Medicine, Imperial College London, London, UK; 3 Department of Diabetes & Endocrinology, University College Hospital, London, UK

## Abstract

**Objective:**

Klinefelter syndrome (KS) is the second-most prevalent chromosomal disorder in men, though late diagnosis is very common and 50–75% of men remain undiagnosed. Evidence suggests that men with KS have impaired quality of life (QoL) but research on how the diagnosis of KS is associated with different QoL domains and what factors influence patients’ QoL is limited. This study aimed to provide a systematic review of the published evidence on factors that influence QoL in men with KS.

**Design:**

Systematic review and meta-analysis with narrative synthesis.

**Methods:**

Medline, Cochrane, Embase, Psychinfo, CINAHL, BASE and relevant publication reference lists were searched in January 2021. Eligible studies included randomised control trials, cohort studies, cross-sectional studies and epidemiology studies on KS and its effect on QoL and all domains of World Health Organisation (WHO) Quality of Life 100 (WHOQOL-100). Clinical studies with no date restriction published in English were included.

**Results:**

Thematic analysis was completed on 13 studies, with a meta-analysis of intelligence quotient completed on 7 studies. Twelve out of the 13 studies suggested that KS negatively affected the QoL outcomes and KS was associated with impairments in physical, psychological, level independence and social relationship domains of WHOQOL-100. Meta-analysis suggested that men with KS have significantly lower full-scale Intelligence Quotient vs controls (*P* < 0.00001).

**Conclusions:**

This is the first evidence synthesis of QoL in men with KS. Current evidence suggests that combined physical and psychological impairments affect men with KS who also experience impairments in relationships and independence in society. Further research is needed to identify factors that influence the QoL in men with KS.

## Introduction

### Rationale

Klinefelter Syndrome (KS) first described by Harry Klinefelter in 1942 (1) is a common aneuploidy in men clinically characterised by small testes, gonadal failure (hypergonadotropic hypogonadism), disrupted spermatogenesis (infertility), gynaecomastia and eunuchoid proportions (arm span exceeds height by ≥7 cm) (2, 3). It affects 1 in 600 men, but 50–75% of men with KS go undiagnosed in their lifetime (2, 4, 5). Almost 90% of men with KS have an XXY karyotype and the remaining 10% have mosaicism (46, XY/47, XXY), higher-grade aneuploidy (48, XXXY; 49, XXXXY), or structurally abnormal X chromosomes (2).

The extent of mosaicism in KS causes an array of cognitive, psychosocial, and physical symptoms which can affect men at varied degrees of severity. These include hypogonadism, gynecomastia, tall stature, small phallus, reduced level of intelligence, depression, autism traits, schizotypal traits and social anxiety which lead to impaired quality of life (QoL) (2, 6, 7, 8, 9). Milder phenotype and lack of distinct dysmorphic features present a real challenge for early diagnosis (3). Testosterone replacement therapy is recommended for patients with KS once serum gonadotrophins begin to rise in early puberty or when serum testosterone levels become hypogonadal (3, 10, 11).

Evidence suggests that patients with KS have more impaired QoL compared to healthy controls; however, research on how the diagnosis of KS affects a patient’s QoL is limited (2, 12, 13).

There is limited knowledge of the various symptoms, outcomes and patient experiences, which may result in health and social inequalities for patients with KS. A greater understanding of the associations between KS and the domains of QoL can better support clinical decision-making and meet the condition-specific needs of patients with KS.

### Objectives

The objective of this study was to conduct a systematic review with meta-analysis to provide new insights and further understanding of QoL in patients with KS and to answer the following research question:

- What is the association between Klinefelter syndrome and the WHOQOL-100 domains/facets of QoL?

### Underpinning framework WHOQOL-100

Due to the many factors influencing QoL in patients with KS, the World Health Organisation (WHO) Quality of Life 100 (WHOQOL-100) was adopted as the overarching framework to underpin this systematic review. The WHOQOL-100 is a validated psychometric scale which can be used to measure QoL as an overall construct and across its six QoL domains: ‘overall QoL’, ‘physical health’, ‘psychological health’, ‘level of independence’, ‘social relations’ and ‘environment’ (14). The subsections were developed by the WHO, by incorporating the important aspects of QoL defined by a range of patients and health professionals from various diseases, specialisms and cultural backgrounds. The WHOQOL-100 is a universal Patient-Reported Outcome Measure (PROM) that can measure individual QoL domains to provide evidence of unmet needs and impaired aspects of QoL.

## Methods

### Protocol and registration

The systematic review followed the Preferred reporting items for systematic reviews and meta-analyses (PRISMA) guidelines for quantitative systematic reviews (15), and the study protocol was registered with PROSPERO (CRD42020173435). The Systematic Review Without Meta-analysis (SWiM) guidelines (16) were adopted for narrative synthesis which was guided by the overarching WHOQOL-100 and its six QoL domains (17). Meta-analysis was conducted where possible by grouping studies measuring overall QoL or outcome measures relating to the domains and facets of the WHOQOL-100 structure. Review manager 5.4 (18) was utilised for this analysis.

### Eligibility criteria and participants

All empirical studies involving male children and/or adults diagnosed with KS and measuring a quantifiable factor of QoL that could be defined within the WHOQOL-100 were reviewed for eligibility. No publication date restrictions were imposed (see Supplementary material, see section on supplementary materials given at the end of this article).

### Search

The search was completed on 21 January 2020 using the following databases: MEDLINE (1946 to present), APA Psychinfo (1956 to present), Embase (1974 to present), CINAHL (1963 to present), Cochrane (2005 to present) and grey search via Bielefeld Academic Search Engine (BASE). A secondary search, using the same strategy, was run covering the period between 21 January 2020 and 20 April 2021 to ensure no recent studies were missed; no new studies were included.

Each database was searched individually, the search keywords included ‘Klinefelter Syndrome’ and MeSH terms ‘48, XXYY Syndrome’, ‘49 XXXXY Syndrome’, ‘XXXY Males’, ‘XXY Syndrome’, ‘XXY Trisomy’ and ‘XXYY Syndrome’ all combined with ‘OR’. Keywords for QoL factors, combined with ‘OR’ included: ‘physical health’, ‘psychological health’, ‘level of independence’, ‘social relations’, ‘environment’, ‘spirituality’, ‘faith’ and ‘personal beliefs’; both groups were then combined with ‘AND’. A full search from CINAHL and Medline is included in the Supplementary material.

### Study selection

**Figure 1:**
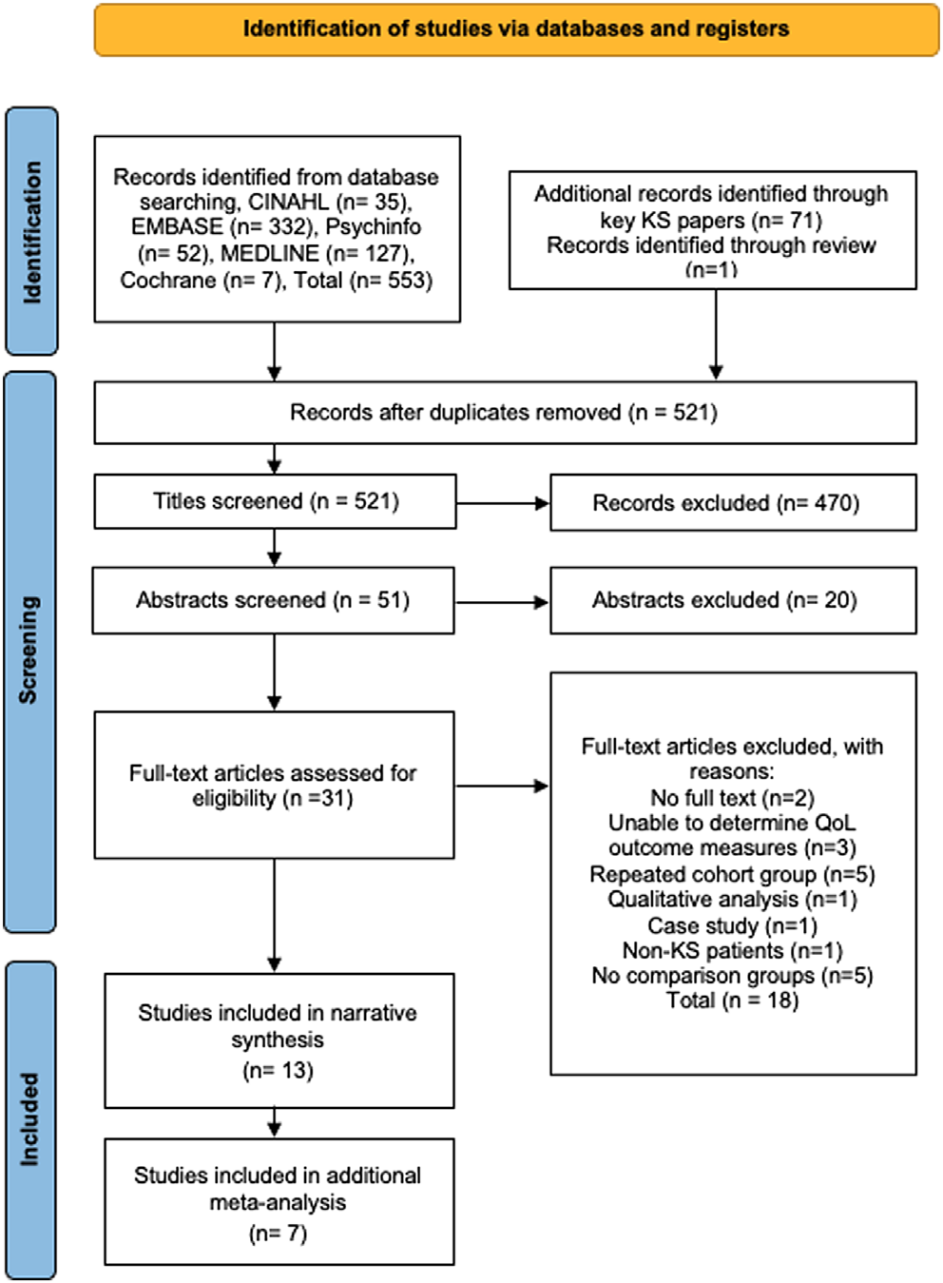
PRISMA flowchart of study search, screening, and selection. A full colour version of this figure is available at https://doi.org/10.1530/EJE-21-1239.

The eligibility assessment was performed in a blind independent review by two authors (BM and SL); all disagreements were resolved by consensus and did not require a third reviewer. The blind review for abstracts and full text articles was done in Rayyan QCRI systematic review manager (https://rayyan.ai/) using pre-specified inclusion/exclusion criteria (Table 1). The PRISMA flow diagram in Fig. 1 annotates the study selection process. A detailed inclusion/exclusion review of all full text articles is included in Supplementary material.

**Table 1:** Characteristics of included studies.

Reference	*n*	Country	Setting	Study design	Methodology	Primary outcome	Measure	Comparator	JB quality score
Ferlin *et al.* (31)	62	Italy	Hospital clinic	Cross-sectional analysis/non-RCT	Interview, self-reported questionnaires	Type of sexual dysfunctions within KS	IIEF-15	60 aged-matched	6/8
Fisher *et al.* (32)	46	Italy	Hospital nits	Cross-sectional analysis	Clinical interviews, psychometric analysis	Prevalence of sexual disorders (GD, paraphilia) in KS	AQ, RME, GIDYQ-AA, SAST, SCL-90-R, IEEF,	43 male controls	7/8
Herlihy *et al.* (21)	87	Australia	Participant home	Cross-sectional analysis	DNA self-admin test, self-administered questionnaires	Psychosocial impact of KS on QoL	PWI, MBSRQ-AS, RSE, K10, short form-1, SIS	General population normative means	7/8
Sorensen (27)	14	Denmark	Schools and clinical setting	Cross-sectional analysis	Clinical examination/ psychological assessment	Physical and mental development in KS	WAIS, school attainment, behaviour rating schedules	19 male controls	6/8
Rapp *et al.* (23)	219	Germany	14 recruitment centres.	Cross-sectional analysis	Medical examination/interviews, (PRO) questionnaires	Measuring QoL in patients with (DSD)	WHOQOL-bref	Healthy European populations	8/8
van Rijn (35)	20	Netherlands	Not specified	Cross-sectional analysis	Self-reported questionnaires, salivary T	Effect of T levels on ‘social anxiety, social cognition’ in KS	FSIQ, KDEF, SCST, SAS	25 male controls	6/8
Skakkebaek *et al.* (33)	132	Denmark	University Hospital	Cross-sectional analysis	Questionnaires, salivary T	Determinants of anxiety and depression	WHOQOL-bref, SF-36, IIEF-15, demographics	313 matched controls	7/8
Skakkebaek *et al.* (34)	69	Denmark	Clinical	Cross-sectional analysis	Questionnaires, cognitive assessments	Cognitive performance in KS	NEO PI-R, AQ, FSIQ	69 matched controls	6/8
Van Rijn *et al.* (7)	34	Netherlands/Belgium	Academic medical clinics /support groups	Cross-sectional analysis	Patient- and parent-reported questionnaires	Social behavioural phenotype in children with KS	ADI-R, SRS, SAS, SSRS	46 male Controls	7/8
Van Rijn *et al.* (26)	31	Netherlands	Not Specified	Cross-sectional analysis	Self-administered questionnaires and tests	Social difficulties in adult men with Klinefelter syndrome	NART, WAIS-R IQ, SIB, Short form, AQ	2 male control groups (*n* = 20, *n* = 24)	6/8
Liberato *et al.*, (22)	58	Italy	Clinical	Cross-sectional analysis	Blood sampling, self-reported questionnaire, and clinical interview	Investigate fluid intelligence, personality traits, personality disorders (PD) in adult KS	SCIDII, MMPI2, SPM	Community samples	5/8
Nielsen & Pelsen (24)	34	Denmark	Clinical	Cohort longitudinal study	Telephone interview	Differences between KS and hypogonadal males with a normal karyotype 46, XY	Job status, Martial status and adoption, Criminality, illness ‘physical or mental’	16 hypogonadal males	6/11
Fabrazzo *et al.* (30)	23	Italy	Academic and medical clinics	Cross-sectional analysis	Questionnaires including pre-existing scales and interviews	Impact of 1 year on TRT on psychopathological recovery and QoL in KS	Q-LES-Q, SCL-90-R, MMSE, TCI-R.	23 matched healthy subjects.	7/8

AQ, Autism Spectrum Quotient (62); ADI-R, Autism diagnostic interview – Revised (73); GSIS, General symptomatic index score; GIDYQ-AA, Gender Identity/Dysphoria Questionnaires for Adults and Adolescents (63); IIEF, International Index of Erectile function (75); K10, Kessler Psychological Distress Scale (66); MMPI-2, Minnesota Multiphasic Personality Inventory 2 (70); MMSE, Minimental State Examination; MBSRQ-AS, Multidimensional body-self relations questionnaire (64); NEO PI-R, Revised NEO personality inventory (67); NART, National adult reading test (81); PWI, Personal Wellbeing index (42); QRI, Qualitative reading inventory (67); Q-LES-Q, Italian Quality of Life Enjoyment and Satisfaction Questionnaire (38); RSE, Rosenberg self-esteem test (65); SI, Social interaction; SAS, Social anxiety scale (78); SF1, short form 1 (61); SF-36, 36 Item short form survey (37); SPM, Standard Progressive Matrices (74); SRS, The Social Responsiveness Scale (80); SAST, Short Anxiety Screening test (76); SCID-II, Structured Clinical Interview for Axis II Disorders (71); SCST, Social cognitive skills test (77); SRSS, Social skills rating scale (79); SCL-90-R, Symptom Checklist-90-R; SLC-ANX/DEP, Subscales of Symptoms checklist 90 Anxiety/Depression (72); TCI-R, Temperament and Character Inventory-Revise; WHOQOL100, World Health Organisation Quality of Life 100 bref (14).

### Data items

Information was extracted from each study on the following: (i) number of participants; (ii) study settings, study country and study design; (iii) outcome measures related to the WHOQOL-100 framework (domains/facets) of QoL such as ‘physical health’, ‘social relations’, ‘psychological’, ‘environment’, ‘level of independence’ or ‘religion/personal beliefs/spirituality’; (iv) comparison groups where possible (Table 1).

### Risk of bias of individual studies

The Joanna Briggs quality appraisal tool for cross-sectional studies/cohort studies (19) was used on all 13 studies included in the systematic review to ensure validity and to examine the reliability with a quantifiable score on each included study. Each question was dichotomised to either YES (1 point) or NO (0 points) producing a scale ranging from 0 (poor quality) to 8 or 10 or 11 (high quality) depending on the appraisal tool. Studies were given an appraisal score depending on how many categories of the appraisal they met: ‘inclusion criteria’, ‘study settings and subjects’, ‘exposure’, ‘confounding factors’, ‘outcomes’ and ‘statistics’ (see Supplementary material).

### Synthesis of results

#### Narrative synthesis

The SWiM reporting items protocol (16) was adopted for the narrative synthesis. Using the WHOQOL-100 framework, subgroup analyses were conducted on each of the six subgroups of the WHOQOL-100 and reported in tables including study, effect size (Cohen’s d) and main findings. A meta-analysis was not possible for all studies due to the large amount of differing and heterogeneous outcome measures and scoring systems for QoL. Therefore, the percentage of significant findings and the strength of the effect sizes are considered within each QoL sub-section.

Data were too heterogeneous for meta-analysis due to the vastly different outcome measures included; therefore, to aid comparability, Cohen’s d was calculated by extracting the mean difference and s.d. from KS and control groups where reported. Accepted categories for Cohen’s d effect sizes as small (0.2), medium (0.5) and large (0.8) were applied (20). Where studies did not report *P* values, these were calculated using Fisher’s exact tests to show any significant differences (*P* < 0.05) between patients with KS and controls.

## Results

### Study characteristics

A total of 665 records were identified from the initial search of which 13 studies, 12 cross-sectional and 1 cohort, met the inclusion criteria and 7 studies were suitable for additional meta-analysis (Fig. 1). The total number of participants across the 13 studies was 829; study sample sizes ranged from 14 to 219 participants*.* Studies had a mixture of patient-reported, parent-reported, or physician-answered questionnaires. Table 1 presents a full summary of the characteristics extracted from each study.

### Quality of studies

The quality of the included studies has an impact on the confidence of findings within the review. First, when assessed many studies did not discuss or include strategies to deal with confounders. Secondly, normative data and population averages were used for controls in three studies which lowers the comparative domain score for those studies (21, 22, 23), Nielsen and Pelsen (24) used hypogonadal males as controls which may also reduce confidence in this study as hypogonadal males QoL outcomes could be reduced due to the symptoms of hypogonadism. Eligible studies were assessed for methodological quality using the Joanna Briggs quality appraisal tools (Table 1).

Sampling methods included non-probability using snowballing (21), self-identification (6, 25, 26), purposive (7, 22, 27, 28, 29, 30) or convenience (12, 24, 31, 32, 33, 34, 35), while one study did not report method of sampling (23).

### Narrative synthesis

#### Overall QoL

Results are reported in Table 2. Three studies measured QoL against controls (21, 30, 33), there was significant difference (*P* ≤ 0.05) between patients with KS and controls for the outcome measures: Personal Well-being Index (PWI) (36), WHOQOL-100 (17), Short Form Survey (Sf-36) (37) and for all quality of life enjoyment and satisfaction questionnaire (Q-LES-Q) (38) subitems. The PWI measures the subjective well-being as the average levels of satisfaction across eight aspects of personal life: (i) health; (ii) personal relationships; (iii) safety; (iv) standard of living; 5(v achieving in life; (vi) community connectedness; (vii) future security, (viii) religious/spirituality. As such, this was included in the overall QoL subgroup analysis. A medium effect size (d = 0.738, d = 0.706) favouring the control group was recorded for PWI (well-being) and PWI (satisfaction) in Herlihy *et al.* (21); it was not possible to calculate effect size in Skakkebaek *et al*. (33). Fabrazzo *et al*. (30) effect sizes were recorded identifying small, medium and large effect sizes favouring the control group (d = 0.471, d = 0.686, d = 1.185) in Q-LES-Q sub items.

**Table 2:** Study results from overall QOL measures, Cohen’s d and findings.

Reference/outcome measure	Effect size ‘Cohen’s d’	Main findings
Herlihy *et al.* (21)		All measures were significantly different between the two groups (P < 0.001). General population and KS, phenotype severity were shown to affect the results of PWI.
PWI		
Wellbeing	0.738	
Satisfaction	0.706	
Rapp *et al.* (23)		
WHOQOL-100		Results for WHOQOL-100 for ranges 0–100 and 4–20 were:
Physical health	0.588	66.4 ± 19.4; 14.6 ± 3.1
Psychological	0.673	63.3 ± 17.8; 14.1 ± 2.8.
Social relations	0.659	59.4 ± 21.9; 13.5 ± 3.5
Environment	0.653	69.9 ± 14.9; 15.2 ± 2.4
Skakkebaek *et al.* (33)		All subscales of QoL ‘WHOQOL-100&SF-36’ showed large significant differences between HC and KS (*P* < 0.001) with the lower scores belonging to KS.
WHOQOL-100	N/A	
SF-36	–	
Fabrazzo *et al.* (30)		All sub-items showed statistical difference (*P* <0.05) compared to HC. Subscales ‘physical health/activities, leisure time activities, social relations, and general activities’ (*P*= ≤ 0.05). No significant differences in subscales ‘Work, household duties, school/class work and subjective feelings.’
Q-LES-Q sub-items		
General life	0.686	
Sexual performance	1.185	
Physical health	0.471	

PWI, Personal Wellbeing index (36); Q-LES-Q, Italian Quality of Life Enjoyment and Satisfaction Questionnaire (38); SF-36, 36 Item short form survey (37); WHOQOL-100, World Health Organisation Quality of Life 100 bref (14).

Rapp *et al.* found significant differences and medium effect sizes in all facets of WHOQOL-100 measured, except for environment, when comparing patients with KS to the reference population (physical health (d = 0.588, *P* < 0.0001), psychological (d = 0.673, *P* < .0001), social relations (d = 0.659, *P* < .0001), environment (d = 0.035, *P* = 0.635)) (23). Similarly, Skakkebaek *et al.* found significant differences (*P* < 0.001) in all domains of WHOQOL-100 between KS patients and healthy controls (33). Fabrazzo *et al.*, when comparing patients with KS post 1-year TRT to healthy controls, found a significant difference of (*P* < 0.05) in all Q-LES-Q sub items. Q-LES-Q subscales had significant differences favouring controls in scales (physical health/activities (*P* = 0.038), leisure time activities (*P* = 0.05), social relations (*P* = 0.003), general activities (*P* = 0.045)).

#### Physical health

Three studies measured outcomes related to physical health against controls (Table 3). Skakkebaek *et al.* found that patients with KS had significantly worse physical health compared to controls (*P* < 0.001) for the following parameters: hypogonadism, gynecomastia, undescended testis, osteoporosis, tremor, varicose veins, pulmonary embolism or leg thrombosis, heart valve disease, dental problem, gingiva, chronic headache, fatigue and anxiety (33). On the other hand, Nielsen and Pelsen’s 20-year cohort longitudinal study found no significant differences in physical health between patients with KS and controls (24). Rapp *et al.* found that patients with KS had significantly lower physical health scores on the WHOQOL-100 (*P* < 0.001) compared to three other groups of patients with disorders of sexual development (DSD): females with congenital adrenal hyperplasia, females with XY- DSD, males with XY-DSD (23).

**Table 3:** Study results from physical health measures, Cohen’s d and findings.

Reference	Outcome measure	Effect size ‘Cohen’s d’	Main findings
Skakkebaek *et al.* (33)	Testicular pain	–	KS experienced significantly more testicular pain than controls, *P* < 0.001. KS also experience significantly less physical activity and were heavier than controls,* P* < 0.001. KS had significantly more comorbidities than controls. *P* < 0.001
	Physical activity	–	
Nielsen and Pelsen (24)	Physical health disorders in the last 10 years	–	There were no significant differences between the XXY and XY groups.
Herlihy *et al.* (21)	SF1- Health status (poor/fair)	–	KS = 34%, general population = 15% to answering poor/fair to health status.

SF1, short form 1 (61).

#### Level of independence

There were limited measures on the level of independence in patients with KS. Work capacity was measured by Nielsen and Pelsen, but no significant differences were found regarding skilled/unskilled labour and unemployment between patients with KS and healthy controls (24).

#### Psychological

A significant difference (*P* < 0.001) was identified between controls and patients with KS in each of these outcomes: autism spectrum quotient (AQ), gender identity/dysphoria, neocriticism, extraversion, conscientiousness, attention switching, imagination, communication, global severity index (GSI), mini mental state examination (MMSE), positive symptom distress index (PSDI), social skills including social behaviour and negative assertion (6, 30, 32, 34). A significant difference (*P* < 0.001) between patients with KS and reference population was also reported by Herlihy *et al.* regarding the psychological measures of body-self relations, self-esteem, sexual identity and psychological distress (21) (Table 4).

**Table 4:** Study results from measures of psychological outcomes, effect size and findings

Reference/outcome measure	Effect size (Cohen’s d)	Main findings
Fisher *et al.* (32)		Adjusted *P* - values between HC and KS were:
AQ	0.822	<0.001
GIDYQ-AA	0.872	<0.001
SCL 90- R* (GSIS)*	0.69	Positive symptom distress index: 0.03, obsession-compulsive: 0.04, somatization 0.03.
Herlihy *et al.* (21)		Significant difference for all psychosocial outcomes measured, when compared with population normative data (P < 0.001).
MBSRQ-AS	0.75	
Appearance evaluation	1.143	
Appearance orientation		
RSE	2.022	
K10	–	K10 found 43% of KS had high/very high psychological distress compared to the general population 10%.
SIS	–	
Sorensen (27)		
Behaviour rating scale	–	*P*-values between KS and controls were (*P* < 0.005) in subscales; intelligence, attention, level of activity. (*P* < 0.05); drive, liveliness. (*P* < 0.025) endurance and interest.
Skakkebaek *et al.* (34)		KS expressed significantly more neuroticism, less extraversion, conscientiousness, and openness to experience (P - values ≤ 0.01), controls scored higher on attention switching, imagination, communication, and social skills, while the scores of patients with KS were more evenly distributed across these scales. Differences between KS and controls for attention switching, imagination, communication, and social skills (*P*< 0.01). Attention-to-detail scores were comparably and normally distributed for both patients with KS and controls (*P* >0 .75)
NEO PI-R		
Neuroticism	1.15	
Extraversion	0.73	
Openness	0.60	
Agreeableness	0.018	
Conscientiousness	0.40	
AQ		
Attention to detail	0.06	
Attention switching	0.58	
Imagination	0.65	
Communication	0.42	
Social skills	0.52	
Van Rijn *et al.* (7)		Total ADI-R score for KS participants was (24.3 ± 15.4), showing that the overall range of ASD symptoms was increased in children with KSe.
ADI-R	–	
Van Rijn *et al.* (26)		AQ score and all subscales were significantly different between controls and KS. KS reported to less frequently display negative assertion, significant difference was (*P* = 0.01).
SIB		
Distress during ‘SI’	1.002	
Frequency during ‘SI’	0.167	
AQ	2.111	
Liberato *et al.* (22)		
SCID-II	–	Detected personality disorders in 31% of the KS sample vs a mean of 10.7% obtained from different community samples.
MMPI-2	–	Showed four altered scales, corresponding to Social Responsibility, Dominance, Ego Strength and Repression, in more than 40% of patients. Twenty-four of 34 MMPI scales were pathological in at least 10% of patients.
SPM	–	The mean raw score was 44 ± 10.8 (10–58), with a maximum score of 60.
Nielsen & Pelsen (24)		
Mental illness diagnosis		There were no significant differences between controls and KS regarding mental illness. However, at the initial examination 41% of KS participants had a mental illness and which was significantly higher than controls (*P* < 0.0021).
Fabrazzo *et al.* (30)		There were statistical differences favouring controls over patients with KS following 1-year TRT in measures of; obsessive-compulsive, anger-hostility, phobias, psychoticism, GSI, PSDI. While MMSE had a much larger statistical difference (*P* = 0.0001). Measures: interpersonal sensitivity, depression, anxiety, PST and TCI-R showed no significant differences between groups.
SCL-90 subscales		
Somatization	0.197	
Obsessive-compulsive	0.870	
Interpersonal sensitivity	0.209	
Psychoticism	0.796	
Anxiety	0.028	
Anger-hostility	0.709	
Phobias	0.675	
Paranoid	0.475	
SCL-90 global- indices		
PST	0.509	
GSI	0.724	
PSDI	1.0	
MMSE	1.490	
TCI-R	–	

AQ, Autism Spectrum Quotient (62); ADI-R, Autism diagnostic interview – Revised (73); GIDYQ-AA, Gender Identity/Dysphoria Questionnaires for Adults and Adolescents (63); K10, Kessler Psychological Distress Scale (66), MMPI-2, Minnesota Multiphasic Personality Inventory 2 (70); MMSE, Mini-mental State Examination; MBSRQ-AS, Multidimensional body-self relations questionnaire (64); NEO PI-R, Revised NEO personality inventory (67); QRI, Qualitative reading inventory (67); RSE, Rosenberg self-esteem test (65); SIB, Scale for interpersonal behaviour (69); SIS, Sexual Identity scale (68); SPM, Standard Progressive Matrices (74); SCID-II, Structured Clinical Interview for Axis II Disorders (71); SLC-ANX/DEP, Subscales of Symptoms checklist 90 Anxiety/Depression (72); SCL-90-R, Symptom Checklist-90-R; TCI-R, Temperament and Character Inventory-Revised.

Fisher *et al.* and van Rijn *et al.* reported significantly greater prevalence of autism symptoms (*P*  < 0.001) as measured by Autism spectrum quotient; both studies and a total of (*n* = 77) participants were included (26, 32). Furthermore, both studies had large effect sizes (>0.8) suggesting there was an association between KS and autism symptoms in these studies.

**Figure 2:**
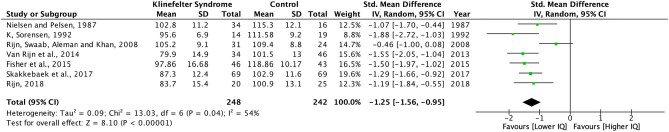
Forest plot comparing Intelligence Quotient scores from KS and Controls from studies measuring domains of quality of life. A full colour version of this figure is available at https://doi.org/10.1530/EJE-21-1239.

A meta-analysis was possible for Intelligence Quotient (IQ) in seven studies, six cross-sectional and one cohort longitudinal (Fig. 2). This included a total of 490 participants across all ages: 248 patients with KS and 242 controls. To measure full-scale IQ, two studies used the Wechsler Adult Intelligence Scale (WAIS) (39), two studies used Wechsler Adult Intelligence Scale – Revised (WAIS-R) (40), one study used the Wechsler Intelligence Scale for Children-III (41), and two studies reported full-scale IQ scores, participants, SD and control data however the (IQ) test used was not listed. For the meta-analysis the study CI and the overall interval was set at 95%. The meta-analysis suggests an association between lower full-scale IQ and a KS diagnosis. There was a strong significant difference between patients with KS and control suggesting a negative association between full-scale IQ for patients with KS when compared to controls. The I2 result (I2 = 54%) showed moderately high heterogeneity (42), which could be due to the varied ages of participants and differing measure of full-scale IQ.

#### Social relations

Eight studies included measures relating to social behaviour, sexual function, sexual satisfaction, and sexuality (6, 7, 26, 28, 31, 32, 35) within the social relations subsection of the WHOQOL-100 (Table 5). With the exception of Turiff *et al.* (6), all studies measuring social relations found that patients with KS have lower scores than their controls.

Four studies (21, 26, 32, 34) compared patients with KS against controls which allowed effect size to be calculated (Table 5).

**Table 5:** Study results from measures of social relations, effect size and findings.

Reference/outcome measure	Effect size (Cohen’s d)	Main findings
Ferlin *et al.* (31)		There was significant difference between KS and controls in sexual desire, intercourse satisfaction, overall satisfaction (*P* < 0.05). Erectile dysfunction (*P* < 0.0005).
IIEF - 15		
Erectile dysfunction	0.385	
Overall satisfaction	0.675	
Fisher *et al.* (32)		KS group showed higher risk of developing hypersexuality and voyeuristic fantasies.
SAST	−0.561	
IIEF		
Overall function	0.706	
Overall satisfaction	0.375	
Van Rijn (35)		The 47, XXY group lower levels of salivary testosterone were significantly associated with higher levels of social anxiety. Salivary levels of testosterone were uncorrelated to social cognitive skills.
SAS	–	
SCST	–	
KDEF	–	
Van Rijn *et al.* (7)		The effect size between healthy controls and KS participants was large in all categories measured, there were significant differences (*P* < 0.05) SRS, SAS.
SAS	0.793	
SRS	2.016	
SSRS	−1.369	
Skakkebaek *et al.* (33)		*P* value <0.001 in orgasmic function, erectile function 0.003, total sexual function 0.008. Intercourse satisfaction 0.006. Parenthood was significantly lower than controls *P* < 0.001.
IIEF		
Overall function	–	
Overall satisfaction	–	
Van Rijn *et al.* (26)		Overall distress during social interactions was significantly higher in the XXY group as compared to men from the general population. Mean score in the XXY group was 2.2 (s.d. 0.67) and in the control group 1.6 (s.d. 0.49), which was significantly different (F (1,52) = 13.2, *P* = 0.001).
Social behaviour	–	
Overall social distress	1.002	

IIEF, International Index of Erectile function (75); SAS, Social anxiety scale (78); SRS, The Social Responsiveness Scale (80); SAST, Short Anxiety Screening test (76); SCST, Social cognitive skills test (77); SRSS, Social skills rating scale (79).

Two studies found that patients with KS had an increased risk in developing negative social traits of anxiety, social responsiveness, and social awareness (28, 29). Tartaglia *et al.* found that more than 25% (*n*  = 42) of patients with KS scored mild, moderate or severe on all domains of the Social Responsiveness Scale (SRS) except for the social awareness domain (28). Furthermore, Van Rijn *et al.* identified a strong effect size (d = 2.016) when measuring social responsiveness using SRS in patients with KS when compared to controls (7).

#### Environment

There were limited measures of ‘environment’, WHOQOL-100 lists the subgroups of ‘environment’ as; financial resources, freedom, physical safety and security, health, and social care: accessibility and quality, home environment, opportunities for acquiring new information and skills, participation in and opportunities for recreation/leisure, physical environment, and transport. Only four studies measured education (21, 24, 25, 33) and two measured financial resources (21, 33).

Acquiring new information and skills can be linked to school attainment and completing education. Herlilhy *et al.* reported that 34% of patients with KS (*n* = 87) did not complete high school, 55% completed high school and 10% studied further than high school (21). Results showed significant differences in five categories: lack of interest in schoolwork (*P* < 0.05), concentration difficulties (*P* < 0.005), speech difficulties (*P* < 0.05), lack of self-confidence (*P* < 0.05), particularly dependent on parents (*P* < 0.025) (21).

Skakkebaek *et al.* found that patients with KS (*n*  = 132) were significantly less likely than controls to complete high school (*P*  < 0.001) and at least 1 year of higher education (*P*  < 0.01) (33). School performance was also significantly worse (*P* < 0.001) for patients with KS when compared to healthy controls (24). Turriff *et al.* looked at the highest education level obtained by patients with KS (*n* = 310) and found that 13.6% completed post-graduate education, 23.9% college, 22.6% part of college education, 13.6% technical school, 22.2% high school and 4.1% completed elementary or junior school (6). Herlihy *et al.* found that 36% of patients with KS (*n*  =87) earned less than AUS$30,000, 27% earned between AUS$30,000 and 69.999 and 30% earned more than AUS$70,000 (21). Similarly, Skakkebæk *et al.* found that patients with KS (*n* = 126) had significantly lower (*P* < 0.001) household income compared to healthy controls (33).

## Discussion

This is the first systematic review to provide an in-depth analysis of the associations between KS and QoL. The WHOQOL-100 provided a framework which allowed sufficient synthesis for many parameters and domains of QoL, which highlighted a disparity in the QoL between patients with KS and controls. Furthermore, the meta-analysis from the included studies indicates a lower full-scale IQ is associated with KS diagnosis.

Almost all patients (95.9% of *n* = 829) across 12 studies included in this systematic review reported that KS had negatively affected the QoL outcome measures. When calculated between patients with KS and controls, a significant effect size (Cohen’s d) was present in most outcomes measured (91.8% of 49). The effect size within this review further quantifies the difference between KS and controls in outcome measures associated with QoL providing hence evidence of the negative impact of the KS diagnosis on patients’ QoL.

Validated measures of QoL, such as PWI (36), WHOQOL-100 (17), Q-LES-Q (38) and SF-36 (37), showed poorer QoL scores for patients with KS compared to controls. This is consistent with previous research which supports overall impaired QoL in patients with KS (13, 43).

Psychological outcomes were the most measured subgroup of QoL and 37 of the 45 outcome measures showed a statistical significance in scores indicating that KS diagnosis is increasing the risk for patients to develop a psychological disorder including cognitive impairment. The results of this systematic review support previous research which found that patients with KS had an increase in psychiatric comorbidities including autism, attention deficit hyperactivity disorder (ADHD), psychosis, personality disorders and developmental disorders (44, 45, 46, 47).

Furthermore, our meta-analysis showed that men with KS have a significantly lower IQ than healthy controls (24, 26, 27, 32, 34, 35) (Fig. 2). Kennedy *et al.* noted the importance of IQ as a predictor of future success (48). Previous research also shows that lower IQ is associated with negative outcomes such as increased prosocial skill deficits, criminal behaviour, post-traumatic stress disorder and lower academic achievements (48, 49, 50, 51, 52, 53). As these outcomes form subgroups of the QoL construct, it is essential to understand the effect that the diagnosis of KS has on the patient’s IQ, in order to provide the necessary care and support at an early-stage post-diagnosis. Further research is necessary to investigate the effect that lower IQ may have on the QoL outcomes for patients with KS.

This systematic review suggests that men with KS are at higher risk than healthy controls to develop psychiatric disorders associated with autism spectrum symptoms, but these are often not recognised or managed appropriately (7, 26, 28, 30, 32, 34). Previous research found that people with autism have more impaired QoL outcomes compared to healthy controls (54), while two studies suggested improved health-related QoL outcomes in people with less severe autism symptoms (55, 56). Like KS, autism has a broad phenotype with a variety of symptoms ranging from disruptive language to socio-emotional traits. However, unlike KS, the awareness and research conducted on autism are far greater which has led to earlier diagnosis and relevant support for patients diagnosed with autism, with improved QoL outcomes and wider social understanding of autism. Evidence supports the presence of autism symptoms especially social behaviours in patients with KS, yet many patients do not receive appropriate investigations nor are diagnosed with autism spectrum disorders which can have a detrimental impact on their QoL outcomes (26, 32, 34).

School attainment and behaviour were measured by five studies which found that boys with KS had significantly lower (*P* < 0.05) achievement and worse behaviours than healthy controls at all levels of education (6, 25, 27, 33, 57). Recent research in education and psychology shows that behaviours and attitudes in school have a direct correlation with work status, income earnings and social status later in later life (58). Although there is limited evidence to support that boys with KS have poor school attainment and behaviours, the consequences of this may have severe lifelong implications. Therefore, further research is needed to investigate this area to develop relevant supportive mechanisms at school for young boys with KS.

This systematic review found that the diagnosis of KS has a significant negative impact on the patients’ erectile function and sexual satisfaction, which is most likely secondary to testosterone deficiency and psychological disorders associated with KS (31). Further research is required to address this problem. Similarly, our review suggests that patients with KS have more increased social anxiety and impaired social skills compared to controls (7, 26, 28, 35). This is supported by two earlier studies which provide evidence of the negative effect that low testosterone has on social anxiety (59, 60).

In conclusion, our systematic review with narrative synthesis and meta-analysis, guided by the WHOQOL-100 as an overarching framework, provides evidence that patients with KS have impaired QoL compared to healthy males. Although evidence for overall QoL outcomes was limited, subgroup analysis helped to provide a greater understanding of the WHOQOL-100 subgroups, and the extent to which each of these are affected by patients with KS. Further research is needed to understand the impact the diagnosis of KS has on patients’ QoL. A significant finding from this systematic review was the lack of a condition-specific PROM for patients with KS. Development and validation of a KS-specific PROM that would encompass all domains of QoL for this patient group and would provide a quantifiable and validated measure for QoL is therefore essential.

## Supplementary Material

Supplementary Material

## Declaration of interest

Sofia Llahana: Occasional scientific consultant and conference fees for Bayer, Novartis, Pfizer and Ipsen. Unrestricted educational grants from Sandoz and Bayer. Senior Editor for Endocrinology, Diabetes and Metabolism Case Reports. Channa Jayasenna: Investigator- led grant by Logixx Pharma Ltd. The other authors have nothing to disclose.

## Funding

No funding has been received for this systematic review. S L is funded by an NIHR Clinical Lectureship. C N J is funded by the Imperial NIHR BRC & NIHR Post-Doctoral Fellowship.

## Author contribution statement

B M and S L conceived the presented idea. B M developed the conceptual framework, theory, methods and searching under supervision of S L, B M and S L completed the blind review of studies. B M completed the collection of data and analysis from the included studies. All authors B M, S L, S G, and C J contributed to the discussion of results and provided critical feedback to all aspects of the manuscript which helped shape the final manuscript. B M, C J and S L completed the abstract. B M and S L took the lead on writing the main body of text.
