# Supplementary material for: Association between domains of quality of life and patients with Klinefelter syndrome: a systematic review
Source: Eur J Endocrinol. 2022 May 31;187(2):S21–34. doi: 10.1530/EJE-21-1239 (PMC9254297; doi:10.1530/EJE-21-1239)
Supplement: Supplementary Material [file supplementary_material.pdf]

Supplementary material

Appendix A: Inclusion/Exclusion criteria.

**Include**	**Exclude**
All quantitative empirical studies	Systematic reviews
Patients with Klinefelter syndrome, all ages	Meta-analysis
Outcome measures related to QoL and WHOQOL-100 dimensions (subsections)	Guidelines, position statements and case studies
Human studies only	Qualitative studies
Full text available	Animal studies
Peer-reviewed published studies	Reviews, editorials and opinion papers
Manuscripts in English	Duplicated cohorts
Studies with comparison groups	Studies without comparison groups

Appendix B: Literature search strategy, CINAHL

Select / deselect all | Search with AND | Search with OR | Delete Searches | Refresh Search Results

Search ID#	Search Terms	Search Options	Actions
S10	S1 AND S9	**Expanders** - Apply equivalent subjects<br>**Search modes** - Boolean/Phrase	View Results (35) | View Details | Edit
S9	S2 OR S3 OR S4 OR S5 OR S6 OR S7 OR S8	**Expanders** - Apply equivalent subjects<br>**Search modes** - Boolean/Phrase	View Results (643,721) | View Details | Edit
S8	spirituality OR religion OR personal beliefs	**Expanders** - Apply equivalent subjects<br>**Search modes** - Boolean/Phrase	View Results (34,917) | View Details | Edit
S7	environment	**Expanders** - Apply equivalent subjects<br>**Search modes** - Boolean/Phrase	View Results (184,599) | View Details | Edit
S6	social relations	**Expanders** - Apply equivalent subjects<br>**Search modes** - Boolean/Phrase	View Results (4,340) | View Details | Edit
S5	level of independence	**Expanders** - Apply equivalent subjects<br>**Search modes** - Boolean/Phrase	View Results (1,216) | View Details | Edit
S4	psychological	**Expanders** - Apply equivalent subjects<br>**Search modes** - Boolean/Phrase	View Results (256,562) | View Details | Edit
S3	physical health	**Expanders** - Apply equivalent subjects<br>**Search modes** - Boolean/Phrase	View Results (49,724) | View Details | Edit
S2	quality of life	**Expanders** - Apply equivalent subjects<br>**Search modes** - Boolean/Phrase	View Results (191,559) | View Details | Edit
S1	klinefelter syndrome OR 48, xxyy syndrome OR 49 xxxxy syndrome OR xxxy males OR xxy syndrome OR xxy trisomy OR xxyy syndrome	**Expanders** - Apply equivalent subjects<br>**Search modes** - Boolean/Phrase	View Results (472) | View Details | Edit

Appendix C: Literature search strategy, MEDLINE

Select / deselect all | Search with AND | Search with OR | Delete Searches | Refresh Search Results

Search ID#	Search Terms	Search Options	Actions
S10	S1 AND S9	**Expanders** - Apply equivalent subjects<br>**Search modes** - Boolean/Phrase	View Results (150) View Details Edit
S9	S2 OR S3 OR S4 OR S5 OR S6 OR S7 OR S8	**Expanders** - Apply equivalent subjects<br>**Search modes** - Boolean/Phrase	View Results (1,592,497) View Details Edit
S8	spirituality OR religion OR personal beliefs	**Limiters** - Date of Publication: -20200131; English Language<br>**Expanders** - Apply equivalent subjects<br>**Search modes** - Boolean/Phrase	View Results (45,355) View Details Edit
S7	environment	**Limiters** - Date of Publication: -20200131; English Language<br>**Expanders** - Apply equivalent subjects<br>**Search modes** - Boolean/Phrase	View Results (744,220) View Details Edit
S6	social relations	**Limiters** - Date of Publication: -20200131; English Language<br>**Expanders** - Apply equivalent subjects<br>**Search modes** - Boolean/Phrase	View Results (7,028) View Details Edit
S5	level of independence	**Limiters** - Date of Publication: -20200131; English Language<br>**Expanders** - Apply equivalent subjects<br>**Search modes** - Boolean/Phrase	View Results (2,031) View Details Edit
S4	psychological	**Limiters** - Date of Publication: -20200131; English Language<br>**Expanders** - Apply equivalent subjects<br>**Search modes** - Boolean/Phrase	View Results (536,843) View Details Edit
S3	physical health	**Limiters** - Date of Publication: -20200131; English Language<br>**Expanders** - Apply equivalent subjects<br>**Search modes** - Boolean/Phrase	View Results (73,599) View Details Edit
S2	quality of life	**Limiters** - Date of Publication: -20200131; English Language<br>**Expanders** - Apply equivalent subjects<br>**Search modes** - Boolean/Phrase	View Results (309,792) View Details Edit
S1	Klinefelter Syndrome OR 48, xxyy syndrome OR 49 xxxxy syndrome OR xxxy males OR xxy syndrome OR xxy trisomy	**Limiters** - Date of Publication: -20200131; English Language<br>**Expanders** - Apply equivalent subjects<br>**Search modes** - Boolean/Phrase	View Results (3,409) View Details Edit

Appendix D: Joanna Briggs Quality appraisal tool: Analytical Cross-Sectional Studies.

Author & year	Were the criteria for inclusion in the sample clearly defined?	Were the study subjects and the setting described in detail?	Was the exposure measured in a valid and reliable way?	Were objective, standard criteria used for measurement of the condition?	Were confounding factors identified?	Were strategies to deal with confounding factors stated?	Were the outcomes measured in a valid and reliable way?	Was appropriate statistical analysis used?
(Ferlin et al., 2018)	Y	Y	Y	Y	N	N	Y	Y
(Fisher et al., 2015)	Y	Y	Y	Y	Y	N	Y	Y
(Herlihy et al., 2011)	Y	Y	Y	Y	Y	N	Y	Y
(K,Sorensen, 1992)	Y	Y	Y	Y	N	N	Y	Y
(Rapp et al., 2018)	Y	Y	Y	Y	Y	Y	Y	Y
(Rijn, 2018)	Y	Y	Y	Y	N	N	Y	Y
(Skakkebaek et al., 2018)	Y	Y	Y	Y	Y	N	Y	Y
(Skakkebaek et al., 2017)	Y	Y	Y	Y	Unclear	N	Y	Y
(Van Rijn et al., 2014)	Y	Y	Y	Y	Y	N	Y	Y
(Van Rijn, Swaab, Aleman and Khan, 2008)	Y	Y	Y	Y	N	N	Y	Y

(Liberato et al., 2017)	Y	Y	Y	Y	N	N	Unclear	Y
(Fabrazzo et al.,2021)	Y	Y	Y	Y	Y	N	Y	Y

Appendix E: Joanna Briggs Quality appraisal tool: Cohort Studies.

Author & year	Were the two groups similar and recruited from the same population?	Were the exposures measured similarly to assign people to both exposed and unexposed groups?	Was the exposure measured in a valid and reliable way?	Were confounding factors identified?	Were strategies to deal with confounding factors stated?	Were the groups/participants free of the outcome at the start of the study (or at the moment of exposure)?	Were the outcomes measured in a valid and reliable way?	Was the follow up time reported and sufficient to be long enough for outcomes to occur?	Was follow up complete, and if not, were the reasons to loss to follow up described and explored?	Were strategies to address incomplete follow up utilized?	Was appropriate statistical analysis used?
(Nielsen and Pelsen, 1987)	Y	N/A	Y	N	N	N/A	Y	Y	Y	N	Y

Appendix F: Full text review. Included, Excluded studies with reasons

#	Author & Year	Is a full text copy of the study available?	Are patients Males with a clinical KS diagnosis?	Is it primary research?<br><br>*If* **YES** *specify the type of study.*	Is the study measuring QOL?<br><br>*If* **YES** *which parameters does it meet e.g. 'Physical health, Environment, Psychological, Social Relations, Level of Independence'*	Can an explicit method or measurement of assessment of these parameters be identified?<br><br>*If* **YES***, specify the measurement of assessment.*	If study is a cohort or an interventional study,<br><br>*What is the comparator?*	Is there an outcome associated with QOL and patients with KS?<br><br>*If* **YES***, what is the outcome?*	Decision:<br>Include or Exclude.<br><br>*If Excluded give reason.*
1.	(Bender et al., 2001)	Y	N - Mix patient group of 23 patients with other genetic conditions, KS not labelled or clinical diagnosis confirmed.	Y - Cross-sectional study	Y - Psychological, Thinking, learning , memory.	Y – Wisconsin card sorting, Woodcock tests. WRAT, WAIS-R tests, WAIRS-R Full-scale IQ	Y - "Controls, including 11 male and 11 female siblings of propositi, were karyotypicaly normal"	Y - "In summary, the presence of an SCA results in increased potential for learning problems in childhood that persist into adulthood"	**Exclude**<br>-KS patients not defined or identified within results.
2.	(Close, Fennoy, Smaldone and Reame, 2015)	Y	Y	Y – Cross-sectional study	Y – Psychological	Y –Questionnaires: Paediatric QOL inventory 4.0 health related- QOL, Piers-Harris 2 self concept scale, Coopersmith self-esteem inventory, Children's depression inventory.	N/A	Y -"Depending on the degree of phenotypic abnormality, boys with KS may be at risk for impaired QOL"	**Exclude**<br>-No comparator
3.	(Ferlin et al., 2018)	Y	Y	Y – Part being assessed Cross-sectional study, second part Non-RCT	Y – Social relations, sexual activity.	Y - 15-Item International Index of Erectile Function, endocrine assessment,	Y - 60 age matched controls.	Y – "KS subjects have lower sexual desire, intercourse satisfaction"	**Include**
4.	(Boratynska-Dabrowsk et al., 1970)	N	N/A	N/A	N/A	N/A	N/A	N/A	**Exclude**<br>– No full text

5.	(Herlihy, 2017)	N	N/A	N/A	N/A	N/A	N/A	N/A	**Exclude**<br>- No full text
6.	(Close,2011)	Y	Y – 43 KS males	Y- Mixed methods (Cross-sectional and retrospective chart review)	Y - Physical health, Psychological	Y – Questionnaires, (Peds QL4.0), Coopersmith self-esteem inventory, Piers-Harris2 short form, (CDI), Physical problems frequencies.	N/A	Y – "Findings of the current study suggest that a high portion of KS (67.4%) have poor total QOL"	**Exclude**<br>-No comparator
7.	(Fisher et al., 2015)	Y	Y	Y- Cross sectional study	Y – Social relations /sexual activity. Psychological/Thinking learning memory and concentration.	Y – Questionnaires. Psychometric tests. (AQ) (RME) (GIDYQ-AA) (SAST) (SCL-90-R) Wechsler Adult Intelligence scale-Revised.	Y- 43 healthy male controls.	Y- "KS is associated with hyper sexuality, paraphilic, and GB, which were mediated by obsessive compulsive and autistic traits."	**Include**
8.	(Herlihy et al., 2011)	Y	Y – Males 18 years and older	Y – Cross sectional study	Y – Psychological, Environment, Social care. Bodily image, Self-esteem, general health, Sexual activity.	Y – (PWI), global life satisfaction, (MBSRQ-AS), Appearance Evaluation, Appearance Orientation subscale, (RSE), (K10), (SIS), (QRI), (Short form-1)	N/A	Y – Shows KS has a significant personal impact.	**Include**
9.	(K,Sorensen, 1992)	Y	Y – 14 males with KS	Y- Cross sectional study	Y – Sexual activity, Psychological/ memory, learning and concentration.	Y – Wechsler Adult Intelligence Scale, School attainment. Mean scores for school difficulties including, Concentration, speech, interest. Experience of coitus and frequency of masturbation.	Y – 19 healthy male controls were used in the studies measuring QOL that have been identified.	Y – "subjects had increased height, reduced weight, impaired hearing, slightly lower intelligence, poor school performance, increased incidence of psychological consultation and lowered sexual activity.	**Include**
10.	(Khan et al., 2018)	Y	Y – 154 KS diagnosed patients amongst mixed cohort.	Y – Cohort study	N	N	N	N	**Exclude**<br>-QOL is not explicitly measured.

11.	(Liberato et al., 2017)	Y	Y – 58 Adult male KS patients	Y – Cross sectional observation	Y – Psychological	Y – Clinical interview and questionnaires measuring. (SCID-ii), (MMPI-2), (SPM).	N/A	Y – PD prevalence was higher than in the general population.	**Include**
12.	(Nielsen and Pelsen 1987)	Y	Y – 34 KS, 16 hypogonadal males without KS diagnosis.	Y - 20 year follow up study.	Y – Psychological, Sexual activity, Level of independence	Y – Anamnestic information and psychopathological symptoms. Occupation status & change in job responsibility,	Y – 16 hypogonadal males without KS diagnosis.	Y – Showed controls and KS patients had better than expected results in the tested measures, however this was post diagnosis and medical intervention for the previous twenty year.	**Include**
13.	(Rapp et al., 2018)	Y	Y – 218 Klinefelter syndrome, other groups in study. KS clearly labelled throughout, where necessary.	Y- Multicentre cross-sectional clinical evaluation	Y – WHOQOL brief	Y – (WHOQOL-100 BREF) is used for KS patients.	Y – Healthy European populations.	Y – The study shows that all conditions included shows a that there’s a significant lower QOL for the patients compared to controls.	**Include**
14.	(Rijn, 2018)	Y	Y- 20 KS boys.	Y – Cross sectional study	Y – Psychological, Intellectual functioning and social anxiety.	Y – IQ, Social Anxiety Scale,	Y - 25, non-clinical controls between 8 and 19 years old.	Y – Mean level of intellectual functioning was significantly lower	**Include**
15.	(Skakkebaek et al., 2014)	Y	Y – 73 KS males were used, Subgroups of Paternal, maternal, skewed and no skewed	Y – Cross sectional study	Y – Psychological,	Y – Autism spectrum Quotient scores. (SCL)	Y – 73 controls	N	**Exclude** -Unable to determine QOL outcomes from study as results cannot be analysed due to subgroup.
16.	(Skakkebaek et al., 2018)	Y	Y – 132 KS males	Y – Cross-sectional study	Y – Psychological, Physical health, Social relations, Sexual activity.	Y – Questionnaires completed by KS patients. (IIEF-15), (WHOQOL-BREF).	Y – 313 matches controls	Y – KS status is associated with lower PQOL and MQOL.	**Include**
17.	(Skakkebaek et al 2017)	Y	Y – 69 KS males	Y – Cross-sectional study	Y – Psychological,	Y – Questionnaires, IQ, (AQ scale), cognitive performance.	Y- 69 Controls	Y- KS is associated with lower intelligence and poorer social skills	**Include**

18.	(Skakkebaek et al., 2018) Anxiety and depression.	Y	N/A	N/A	N/A	N/A	N/A	N/A	**Exclude**<br>-Results in table one are identical to table one in, (Skakkebaek et al.,2017) The role of genes.
19.	(Tartaglia et al., 2010)	Y	Y – 57 Children and adolescents with KS	Y – Cross sectional study	Y – Psychological,	Y – Questionnaires, Including (WISC-IV), (WASI), (SRS), ADHD symptoms, (ADI-R), (ADOS),	N/A	Y – Diagnosis of KS support having greater prevalence of psychological conditions and cognitive function.	**Exclude**<br>-No comparator
20.	(Temple and Sanfilippo,2003)	Y	Y – 3 KS	Y – Case study	N/A	N/A	N/A	N/A	**Exclude**<br>-Case study
21.	(Turriff, Levy and Biseecker, 2011)	Y	Y- 310 self reported XXY aged 14-75	Y – Cross sectional study	Y – Psychological	Y – Questionnaire using online websites. (CES-D),	N/A	Y – KS may be at increased risk for depressive symptoms.	**Exclude**<br>-No comparator
22.	(Turriff, Levy and Biesecker,2015)	Y	Y- 310 self reported XXY aged 14-75	N/A	N/A	N/A	N/A	N/A	**Exclude**<br>-Participation cohort seemingly used for (Turriff, Levy and Biseecker,2011)
23.	(Turriff, Macnamara, Levy and Bisecker, 2016)	Y	Y- 310 self reported XXY aged 14-75	Y – Thematic analysis.	N/A	N/A	N/A	N/A	**Exclude**<br>-Thematic analysis
24.	(Van Rijn and Swaab, 2011)	Y	Y – 73 Ks subjects	Y – Cross sectional study.	Y – Psychological,	Y- Wechsler Intelligence scales, Autism questionnaire, Schizotypal personality Questionnaire.	Y – 93 age matched controls	Y – Psychopathology traits were significantly higher with KS than the control group.	**Exclude**<br>-Patient group recruited from same pool as (Rijn et al., 2008 & Rijn et al 2006).

25.	(Van Rijn and Swaab,2015)	Y	Y – 23 KS boys, clearly labelled, other participants 17 girls with Trisomy X, XXX.	Y – Cross sectional study	Y – Psychological	N– Outcome measures not shown for KS males	Y – 100 non clinical controls, 47 boys and 53 girls.	Y – Executive function is impacted from an additional chromosome affecting everyday function.	**Exclude**<br>-outcome measures
26.	(Van Rijn et al., 2014)	Y	Y – 34 boys with KS and 26 girls with Trisomy X	Y – Cross sectional study	Y- Psychological, social relations	Y – IQ, (SSRS), (SRS), social anxiety.	Y – 106 non clinical controls 46 boys and 60 girls.	Y – Score between boys and girls were similar with an extra X, scores showed more prevalence in the test than the controls. Differences between boys and girls shown, in results.	**Include**
27.	(Van Rijn et al., 2016)	Y	Y – 29 KS boys and 21 Trisomy X girls. Various results explain both girls and boys results.	Y – Cross sectional study	Y – Psychological, thinking, learning and memory.	Y – IQ, Cognitive performance scores,	Y – 103 non-clinical controls.	Y – Intellectual functioning was lower in the KS group than controls. Early life stress has a greater negative on those with an extra chromosome than those without.	**Exclude**<br>-Same patient group used as (Van Rijn et al., 2013)
28.	(Van Rijn, de Sonneville and Swaab, 2018)	Y	Y- 70 males with KS.	Y – Cross sectional study	Y – Psychological, thinking, learning and memory. Social relations	Y – Wechsler Intelligence scales, Full scale IQ:FSIQ), (ANT),	N/A	Y – KS is linked to lower social cognition with regards to ToM, and fits other studies measuring similar outcomes.	**Exclude**<br>-No comparator
29.	(Van Rijn, Swaab, Aleman and Khan, 2006)	Y	Y – 32 KS men	Y – Cross sectional study	Y – Psychological, Social relations	Y – (Raven's Advanced Progressive metrics, short form), (NART), Benton and Van Allen Test,	Y - 26 men from the general population.	Y – KS had greator social cognition disturbances than that of the general population.	**Exclude**<br>-Similar patient group from same participant pool as,(Van Rijn, Swaab, Aleman and Khan,2008).
30.	(Van Rijn, Swaab, Aleman and Khan, 2008)	Y	Y – 31 KS men	Y – Cross sectional study	Y – Psychological, Social relations	Y – (Ravens Advanced Progressive metrics, (NART), scale for interpersonal behaviour(SIB), (Autism spectrum Quotient),	Y – two groups of 24 and 20 male controls.	Y – KS men had increased distress in social situations and less engagement in certain social situations. Ks also had significant higher autism traits.	**Include**

31	(Fabrazzo et al., 2021)	Y	Y – 23 Men with KS post 1-year androgen therapy.	Y – Cross-sectional study	Y – Overal and psychological measures	Y – Q-LES-Q, MMSE, SCL—90-R, TCI-R	Y – 23 healthy controls	Y – KS patients, treated for one year with testosterone undecanoate, do not yield a QoL compared to matched healthy controls.	**Include**
